# Prevalence of Unmet Care Needs of community based People with Dementia and their caregivers in Lusaka

**DOI:** 10.1002/alz.088746

**Published:** 2025-01-09

**Authors:** Faith Simushi, Jeremy A. Tanner, Stanley Zimba, Deanna Saylor

**Affiliations:** ^1^ University Teaching Hospital, Lusaka, Lusaka Zambia; ^2^ Biggs Institute for Alzheimer’s and Neurodegenerative Disease, University of Texas Health San Antonio, San Antonio, TX USA; ^3^ Johns Hopkins Global Neurology, University Teaching Hospital, Lusaka, Lusaka Zambia

## Abstract

**Background:**

Globally, 47.5 million people were living with dementia in 2015. This figure is expected to reach 75.6 million in 2030, and over 135 million by 2050, with Sub‐Saharan Africa being among the regions expected to have the greatest future increase in dementia incidence and prevalence due to the aging population. With this growing number across the world, identifying a source of regular caregiving for people with dementia (PWD) has become a routine practice. While high income countries have formal support systems for people living with dementia and their caregivers, including adult day care and residential programs, in lower‐income countries such as Zambia, informal caregivers (often family members) assume this responsibility and serve as an interface between PWD and health services. The challenges and needs of community‐based PWD and their caregivers in Zambia have not previously been identified and understood; hence limiting the medical team’s ability to address those needs and increases the risk of negative outcomes for PWD.

**Method:**

This was a cross sectional study (n = 33) community based people living with dementia each with a caregiver. The participants underwent a one‐time in‐depth in‐home assessment enabling collection of socio‐demographic characteristics, clinical characteristics, measurement of performance in activities of daily living and impact of supporting someone with dementia. Assessment of care needs was a based on the John Hopkins Dementia Care Needs Assessment tool. Regression analyses were conducted to identify demographic, clinical, functional and quality of life correlates of unmet needs as well as identify the independent predictors of unmet needs among PWD and their informal caregivers

**Result:**

The mean proportion of unmet needs for patients with dementia was 57% while that of caregivers was 62%. Significantly higher unmet needs were associated with having attained a tertiary education and having an unknown type of dementia.

**Conclusion:**

The study identified that patients with dementia and their informal caregivers had a high prevalence of unmet care needs in multiple domains. It also demonstrated that in this particular cohort, having a tertiary education and an unknown type of dementia was significantly associated with a higher proportion of unmet needs.

